# Transcriptomic profile investigations highlight a putative role for NUDT16 in sepsis

**DOI:** 10.1111/jcmm.17240

**Published:** 2022-02-17

**Authors:** Susie Shih Yin Huang, Darawan Rinchai, Mohammed Toufiq, Basirudeen Syed Ahamed Kabeer, Jessica Roelands, Wouter Hendrickx, Sabri Boughorbel, Davide Bedognetti, Nicholas Van Panhuys, Damien Chaussabel, Mathieu Garand

**Affiliations:** ^1^ Division of Pediatric Cardiothoracic Surgery, Department of Surgery Washington University School of Medicine St Louis Missouri USA; ^2^ Sidra Medicine Doha Qatar; ^3^ College of Health and Life Sciences Hamad Bin Khalifa University Doha Qatar

**Keywords:** ADP‐ribosylation, gene expression, mRNA decapping, nudix hydrolase, nudix hydrolase 16 (NUDT16), reductionist approach, sepsis

## Abstract

Sepsis is an aberrant systemic inflammatory response mediated by the acute activation of the innate immune system. Neutrophils are important contributors to the innate immune response that controls the infection, but harbour the risk of collateral tissue damage such as thrombosis and organ dysfunction. A better understanding of the modulations of cellular processes in neutrophils and other blood cells during sepsis is needed and can be initiated via transcriptomic profile investigations. To that point, the growing repertoire of publicly accessible transcriptomic datasets serves as a valuable resource for discovering and/or assessing the robustness of biomarkers. We employed systematic literature mining, reductionist approach to gene expression profile and empirical *in vitro* work to highlight the role of a Nudix hydrolase family member, NUDT16, in sepsis. The relevance and implication of the expression of NUDT16 under septic conditions and the putative functional roles of this enzyme are discussed.

**Abbreviations**
ADP: adenosine diphosphate
DCP1: mRNA‐decapping enzymes
GEO: Gene Expression Omnibus
GXB: Gene Expression Browser
IL‐6: interleukin 6
LPS: lipopolysaccharide
NETs: Neutrophil extracellular traps
PGN: peptidoglycan
TNFα: tumour necrosis factor alpha

## 1 INTRODUCTION

Sepsis is an aberrant systemic inflammatory response mediated by the acute activation of the innate immune system.^1^ The disease currently affects 19 million patients worldwide, with the mortality rate between 25% and 30%.^2^ Sepsis can clinically deteriorate into shock with the appearance of organ dysfunction and refractory hypotension. During sepsis, the innate immune system is activated by the recognition of pathogen‐derived molecules via receptors expressed on a wide range of host cells. Receptor activation causes the release of soluble inflammatory mediators, and microbicidal molecules. Neutrophils are important contributors to the innate immune response that controls infections but can also lead to considerable collateral tissue damage,^3^ such as neutrophil extracellular traps (NETs)‐related thrombosis and organ dysfunction.^1^ Thus, a better understanding of how cellular processes in neutrophils and other contributing leukocytes are modulated during sepsis is needed.^4^ Such an endeavour can be initiated via the investigation of transcriptomic expression profiles. To that point, the growing repertoire of publicly accessible transcriptomic datasets, like those on the Gene Expression Omnibus (GEO), serves as a valuable resource for discovering novel predictors, diagnostic markers and disease progression markers. Diverse reductionist approaches have been used to successfully identify putatively novel genes/functions^5^, ^6^, ^7^, ^8^, ^9^ and perform system‐level re‐analyses.^10^, ^11^, ^12^

The anti‐inflammatory and immunosuppressive state of late‐phase sepsis resemble endotoxin tolerance in which the TLR4‐dependent pathway is non‐responsive to gram‐negative bacterial lipopolysaccharide (LPS; reviewed in^13^). The underlying molecular mechanisms are centred on mRNA‐decapping enzymes (DCP1) and the assembly of the translational repressor complex, which facilitate the degradation of TNFα and IL‐6 mRNA and the formation of p‐bodies in monocytic cells.^14^ NUDT16 is a member of the Nudix hydrolase family and catabolizes nucleoside triphosphates, non‐nucleoside polyphosphates and capped mRNAs,^15^ initiating RNA turnover.^16^ NUDT16 also possesses important DNA protective roles and can impact mediators of DNA repair.^17^

In this study, *NUDT16* was identified as a suitable candidate gene for a reductionist investigation of transcriptomic profiles based on the following criteria: 1) being consistently upregulated in septic patients compared with healthy individuals across multiple transcriptomic datasets, and 2) the absence of overlap between NUDT16 and sepsis in the current literature.

## 2 MATERIALS AND METHODS

### 2.1 An exploratory reductionist approach

Public repository of articles and data, such as PubMed and GEO, represents a vast resource but can be complicate to explore. Here, we present a logical reductionist approach to investigate putative novel biomarkers in sepsis. The approach can be applied to any field of research.

The steps consist of (1) identifying a gene of interest based on its differential expression in the pathological/physiological context of interest, (2) validating the reproducibility of the initial observation, (3) determining the current body of literature linking the gene and topic, (4) extracting the known biological concepts regarding to the gene and (5) inferring putative novel roles to the gene with literature support.

### 2.2 NUDT16 expression in blood cells

A dataset that we have previously generated and deposited in GEO (GSE60424) was used to assess the expression of *NUDT16* across leukocyte populations. The dataset consists of RNA‐Seq profiles of neutrophils, monocytes, B cells, CD4+ T cells, CD8+ T cells and NK cells isolated from blood of healthy controls, patients with type 1 diabetes, amyotrophic lateral sclerosis, sepsis or multiple sclerosis prior to and 24 hours post treatment with interferon beta (up to 20 subjects/cell type). *NUDT16* expression profile is accessible for this dataset via the GXB data browsing web application (link). Significant differences (*p* < 0.0001) were determined by one‐way ANOVA with Tukey's multiple comparisons test.

### 2.3 PubMed queries for NUDT16 literature

The NUDT16 literature was retrieved using a PubMed query which comprised of its official symbol, name and known aliases: ‘Nudix hydrolase 16’ [tw] OR NUDT16 [tw] OR H29K [tw].

### 2.4 Independent datasets for concordance

Datasets were obtained from GEO to be used to conduct independent validation of the initial finding in a relevant clinical setting. Validation data were selected without a priori knowledge of *NUDT16* expression levels and consists of only human studies in which transcriptome profiles were generated in septic patients and compared with uninfected controls (Table 1). Other information was retrieved from each GEO entry, such as the geographic locations of the patient populations under study which spanned four continents, and the type of biological samples, which included purified neutrophils, PBMCs, and whole blood. The studies included neonate, paediatric and adult populations. Finally, these data were generated using two different microarray platforms. Further sepsis‐related datasets can be found and interactively explored in the database recently created by our group, SysInflam HuDB (link).^18^

**TABLE 1:** Description of the validation dataset and statistical results for *NUDT16* expression under septic conditions

GSE ID (GLP ID)	Study Title (sample number)	Age category	Tissue type	Transcriptomic platform	Origin of samples	Group A	Group B	Exp A	Exp B	B/A	*t*‐test	*F*‐test
GSE30119	… whole blood transcriptional response to community‐acquired Staphylococcus aureus infection … (143)	Paediatric	Whole Blood	Illumina	USA	Healthy	*S. aureus*	37.5	62.4	1.7	<0.001	<0.001
GSE16129 (GPL97)	Enhanced Monocyte Response & Decreased Central Memory T Cells in Children with Staphylococcus aureus Infection (52)	Paediatric	PBMCs	Affymetrix	USA	Control	*S. aureus*	716.2	960.3	1.3	<0.01	<0.01
GSE64457	Marked alterations of neutrophil functions during sepsis‐induced immunosuppression (23)	Adult	Neutrophils	Affymetrix	France	Control	Septic patient	36	57.5	1.6	0.053	<0.05
GSE13015	…A Blood Biomarker Signature for the Diagnosis of Septicemic Melioidosis‐ (39)	Adult	Whole Blood	Illumina	Thailand	Healthy & T2D	Melioidosis & other sepsis	39.9	84	2.1	<0.001	<0.001
GSE25504 (GPL6947)	Whole blood mRNA expression profiling of host molecular networks in neonatal sepsis (63)	Paediatric	Whole Blood	Illumina	UK / Gambia	Control	Infected	125.7	162.1	1.3	<0.001	<0.001
GSE72829	Diagnosis of childhood bacterial and viral infection using host RNA expression (104)	Paediatric	Whole Blood	Illumina	UK	Control	Bacteremia	13.7	27.4	2.9	<0.001	<0.001
GSE64456	Defining RNA Transcriptional Biosignatures to Distinguish Febrile Infants 60 Days of Age and Younger with Bacterial vs Non‐Bacterial Infections (44)	Neonate	Whole Blood	Illumina	USA	Control	Bacteraemia	25.1	86.9	3.5	<0.001	<0.001

### 2.5 *In vitro* stimulation assay

Heparinized diluted whole blood (WB) from healthy donors was exposed to media (control) or combined LPS/PGS at 100 ng/ml and 5 ug/ml, respectively, to simulate septic conditions. Samples were collected at 6 h post‐stimulation for total RNA and overnight for protein expression analyses. Briefly, whole blood (maximum total of 4 ml) from each donor was collected via venipuncture into a heparin‐sulphate vacutainer (Becton Dickinson) and mixed at a 1:1 ratio with RPMI (Gibco). 500 ul of WB:RPMI mixture was added to cryovial or microwell already containing the specific stimulation. The samples were incubated in tissue culture incubator at 37°C with 5% CO_2_ for the desired time. For transcript abundance, the 6 h incubation was optimally selected based on previously reported time‐course data for *NUDT16* expression (GSE3284).^19^ The experiment was repeated twice.

### 2.6 RNA extraction and qPCR

At the time of collection, cultured whole blood samples were mixed with PAXgene™ reagent (Qiagen), at a sample‐to‐reagent ratio of 1:2.76, and then gently inverted and stored at −80°C within two hours. Total RNA extraction was performed with PAXgene™ Blood RNA Kit (Qiagen) according to manufacturer's protocol. cDNA was synthesized from 500 ng of total RNA using SuperScript™ III First‐Strand Synthesis System (Invitrogen) and analysed by qPCR using SYBR Green PCR Master Mix (Thermo Fisher) and on a QuantStudio (Thermo Fisher) with the following thermal cycles: initial denaturation at 95°C for 10 min; 40 cycles of denaturation at 95°C for 15 s and annealing/extension at 60°C for 1min. For Melt curve analysis, the following thermal parameters were used as follows: (1) 95°C for 15 s, (2) 60°C for 1 min, (3) 95°C for 15 s and (4) 4°C, hold. Transcript of interest (RefSeq accession number in parentheses) was detected using the following primer pairs (F: forward, R: reverse): *NUDT16* (NM_152395.3) ‐ F: 5’‐TTTCTCCTCCTGTTAGCCATTC‐3’, R: 5’ CCCTACCTGGCTCTTCTATACT‐3’: *GAPDH* (NM_002046.7) ‐ F: 5’‐GAAGGTGAAGGTCGGAGTC 3’, R: 5’‐GAAGATGGTGATGG GATTTC‐3’. Target gene expressions were normalized to GAPDH expression and are shown relative to the control samples (ΔΔCt method). The experiments were performed in duplicates and repeated twice.

### 2.7 Flow cytometry

Cultured whole blood phenotyping and immunostaining for surface and intra‐cellular markers were performed using the standardized protocols published by Cytobank.^20^ Briefly, cells preserved frozen in FACS Lysing Solution (Becton Dickinson) were thawed for 15 min in a 37°C water bath and washed twice in stain buffer (Becton Dickinson, BD). Samples were then permeabilized with Phosflow Perm Buffer II (BD), washed twice in stain buffer, incubated with FC block (cat# 564220, BD) for 10 min at room temperature and stained by adding Normal Goat Serum (10 ul, cat#0060‐01, Southern Biotech) and the recommended volume of antibodies. Fluorescence‐minus‐one controls were also prepared for each fluorochrome. After a 60 min incubation at room temperature in the dark, cells were washed twice. Compensation controls were prepared using Ultracomp eBeads (Invitrogen) following the same preparation as described above. Post‐staining, all samples were then fixed in 4% PFA (Invitrogen), washed twice and resuspended in stain buffer prior to flow cytometric analysis. The fluorochrome‐labelled antibodies used were CD45 V500 (HI30, RRID:AB_1937324, BD), HLA‐DR Pacific Blue (L243, RRID: AB_2561913, BioLegend), rabbit anti‐human NUDT16 primary (Human Protein Atlas Number:HPA011252, Sigma/Merck), Goat anti‐rabbit superclonal secondary antibody Alexa 488 (RRID:AB_2536097, Thermo Fisher), Fixable viability Dye (FVD) UV495 (cat# 423107, BioLegend), CD11b BUV661 (D12, RRID:AB_2874279 BD), CD16 PE (3G8, RRID:AB_2563801, BioLegend), CD11c PE‐Dazzle594 (3.9, RRID:AB_314176, BioLegend), CD3 PE‐Cy7 (UCHT1, RRID:AB_2738196 BD) and CD66b APC‐Cy7 (G10F5, RRID:AB_2750184, BioLegend). Acquisition was performed on a BD Symphony A5 (Becton Dickinson). Compensation beads were used to standardize the voltage settings and used as the single‐stain positive and negative controls. A minimum of 100,000 uncompensated events were acquired from each sample and compensation was set in FlowJo V.10 (FlowJo Tree Star). Gating during analysis (Figure S1A) was based on the fluorescence‐minus‐one principle. Cell viability was assessed via Forward and Side Scatter (FSC/SSC) appearance as previously described^21^, ^22^ or with Zombie UV Fixable Viability kit (BioLegend). The experiments were performed in duplicates and repeated twice.

### 2.8 Statistical analyses

GraphPad Prism v.5 (Graphpad Prism Software) was used for all plots and statistical analyses. Outliers determined by removal of outliers (ROUT) method (Q = 1%) and remove from median calculation. The Mann–Whitney test was used to identify significant differences between groups.

## 3 RESULTS

**FIGURE 1:**
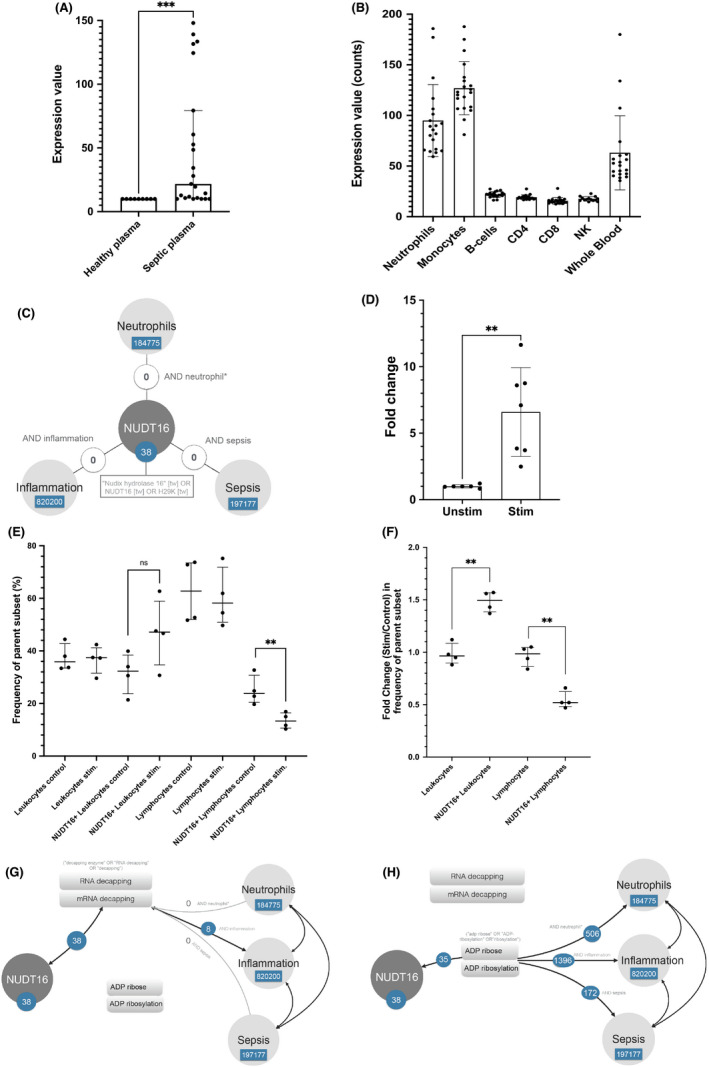
NUDT16 gene and protein expression in septic conditions and extraction of biological concepts associated with NUDT16 from the literature. (A) Expression levels of the Nudix Hydrolase family member 16 gene (*NUDT16*) after *in vitro* exposure of neutrophils to control or septic serum (data from GSE49755). (B) NUDT16 gene expression (expression values, counts) in various immune cell subtypes obtained from dataset GSE60424. (C) PubMed query for NUDT16 and inflammation, neutrophils or sepsis‐related literature. No overlaps were found, indicating a probable biomedical knowledge gap. (D) Whole blood from healthy donors (*n* = 7) post 6h with LPS/PGN or culture medium (Control). *NUDT16* expression was assessed by RT‐qPCR and normalized to *GAPDH* transcript expression. (E) Expression of NUDT16 in cultured whole blood immune cells before and after stimulation for 24 h (expressed as % of parent cell population). (F) Relative abundance of NUDT16 in cultured whole blood expressed as the fold change in frequency parent cell population (stimulated/non‐stimulated). G‐H) Intermediate biological concepts linking indirectly NUDT16 to neutrophils, inflammation or sepsis literature. The most prevalent biological concepts returned were then used in searches against the neutrophil, inflammation or sepsis literature. The extent of the overlap with the NUDT16 literature is shown for the two dominant concepts, ‘RNA decapping’ and ‘ADP ribosylation’

We identified *NUDT16* as the candidate gene based on its expression in healthy neutrophils exposed to plasma from septic patients compared with healthy individuals. This observation was made in a dataset generated in our previous work, which is publicly available in GEO under accession GSE49755,^23^ and can be visualized on the Gene Expression Browser (GXB, link). In brief, the study was conducted in the Northeast of Thailand and involved adult subjects who were hospitalized with symptoms of sepsis and showed culture positive results for either *Burkholderia pseudomallei*, the etiological agent of melioidosis, or other bacterial species. *In vitro* cultures of neutrophils isolated from blood of two healthy donors were exposed to serum samples fractionated from blood of healthy controls (*n* = 6) and individuals hospitalized with bacterial sepsis (*n* = 12). After 6 h of exposure to the serum, neutrophils were lysed, and total RNA extracted for profiling on the Illumina HT12 Bead arrays. We downloaded the data and processed them for outlier identification (ROUT method). *NUDT16* expression profile (Figure 1A) showed significant differences in both the expression levels (4.9‐fold change) and variance between septic and control serum treated groups (*t*‐test *p* < 0.005, *F*‐test <0.001).

We found that *NUDT16* is expressed in several immune cell types with the highest expression level in granulocytes and monocytes, suggesting a greater contribution from these cell types to the expression measured in whole blood (Figure 1B; GSE60424, further details provided in *Materials and Methods* and at this GXB link). This expression pattern among blood cells is further supported by data from the Human Protein Atlas (Figure S1B).

To summarize the current knowledge about NUDT16 in the context of sepsis, we conducted a PubMed query that comprised of its official symbol, name and known aliases (see details in *Materials and Methods*). A total of 38 articles was returned as of 6 January, 2022. However, we found no overlaps between NUDT16 literature with sepsis, inflammation or neutrophils (Figure 1C). Expanding the search using ‘Nudix hydrolase family’ returned one overlapping article with neutrophils and sepsis each but none with inflammation (Figure S2A). The results indicate that there is no known role for NUDT16 in the context of sepsis, inflammation or neutrophil immunobiology in the current published literature.

The robustness of the increase in *NUDT16* transcript abundance in the context of sepsis can be further assessed in a range of settings and methodologies. Indeed, we explored the expression pattern of *NUDT16* during sepsis in seven additional public datasets (see detailed step in *Materials and Methods*) composed of different tissue matrixes and cohorts (summarized in Table 1). Significant (*p* < 0.01) increases in *NUDT16* expression in septic compared with non‐septic patients were observed in six datasets (Table 1, select datasets presented in Figure S2B). Differences in the magnitude of the change may be attributed to the *in vivo* design and the mixed cellular populations in whole blood or peripheral blood mononuclear cells. Additional sepsis‐related datasets are available for rapid and interactive exploration in *SysInflam HuDB* (Link).^18^ Using *SysInflam HuDB*, we found that *NUDT16* expression was consistently increased in patients with shock (GSE57065, GSE66099 and GSE95233), and was higher in bacterial infections compared with other types of infections (e.g. viral) (GSE6269, GSE64456 and GSE30119). Melioidosis patients also exhibited higher *NUDT16* expression compared with other causes of severe infection (GSE69528, GSE13015).

We then set up *in vitro* experiments to have a closer look at *NUDT16* expression in whole blood. We measured the *NUDT16 levels* in cultured whole blood (obtained from healthy adult volunteers, *n* = 7) exposed to a combination of lipopolysaccharide (LPS) and peptidoglycan (PGN) or culture medium (control); we observed a 6.6‐fold increase in *NUDT16* expression with stimulation to unstim (Figure 1D). A portion of the cultured whole blood samples mentioned above was analysed using multi‐parameter flow cytometry. We observed contrasting NUDT16 abundance—we saw a 1.5‐fold increase in the frequency of NUDT16+ leucocytes but a significant 1.85‐fold decrease in the frequency of CD3+ T lymphocytes relative to the change in the respective cell population with stimulation (Figure 1E). These trends were consistent among the different individual donors, despite the different baseline frequencies (Figure S2C). Hence, the calculated fold changes (stimulation/control) showed significance for both NUDT16+ leukocytes and lymphocytes compared with their respective parent populations (Figure 1F); representative dot plots of the relative frequency of NUDT16+ cells before and after stimulation are shown in Figure S2D. The intrinsic level of NUDT16 in other identifiable cell subsets did not show significant changes with stimulation and was not included in the analyses (data not shown).

To gain a general overview of the biological functions of NUDT16, the literature mining approach was re‐utilized. Biological concepts were manually extracted from the NUDT16 literature by using the NUDT16 query presented earlier, but this time restricting the search to title by using [ti] (instead of [tw] = ‘text words’). This search returned 10 articles. Keywords were identified and clustered using term frequency–inverse document frequency (TF‐IDF) and cosine distance function of Document Clustering package in R (details available at cran.r‐project.org) and summarized on the basis of their related biological concepts (Table 2). Next, the prevalence of these concepts in the overall NUDT16 literature was determined using PubMed queries for NUDT16 (text words [tw]) and each concept individually. The largest number of PubMed hits recorded was for the keyword ’ADP‐ribose/ribosylation’ with the following query: *(‘Nudix hydrolase 16’ [tw] OR NUDT16 [tw] OR H29K [tw]) AND (adp OR bp OR nudix OR proteins OR ‘adp ribose’ OR ‘ADP*‐*ribosylation’ OR ribosylation*) (38 articles). When searching for *‘Human NUDT’ or ‘RNA decapping’*, 35 and 38 articles were returned, respectively; combining these two concepts returned the same 38 articles as for *‘ADP*‐*ribose*/*ribosylation’*. Using the same strategy, the concept *‘Cell growth and arrest’* returned 15 articles.

**TABLE 2:** Clusters of biological concepts identified

cluster	top_words	Reduced term	PubMed Articles
1	adp, bp, nudix, proteins, adp_ribose	ADP‐ribose/ribosylation	38
2	human, human_nudt, nudt, activity, activity_human	Human NUDT	38
3	rna, decapping_enzyme, rna_decapping, decapping, enzyme	RNA decapping	35
4	growth, accumulation, accumulation_single, arrest, breaks	Cell growth and arrest	15

To determine the relevance of RNA/mRNA decapping and ADP‐ribosylation in the sepsis, inflammation or neutrophil literature, a series of PubMed queries were performed, for example: *(‘decapping enzyme’ OR ‘rna decapping’ OR ‘decapping’*) AND inflammation identified 8 articles linking indirectly NUDT16 with inflammation (Figure 1G). No articles were found among the sepsis or neutrophil literature that relates specifically to RNA decapping. Similar queries were ran against the inflammation, sepsis or neutrophil literature for ADP ribosylation *(‘adp ribose’ OR ‘ADP*‐*ribosylation’ OR ‘ribosylation’*) and returned 1396, 172, 506 articles respectively (Figure 1H).

## 4 DISCUSSION

The NUDT16 protein is primarily associated with *‘*RNA decapping*’* and ‘ADP‐ribose/ribosylation’; those concepts were presented in 35 and 38 of the articles retrieved. The conceptual link between decapping activity and sepsis/inflammation is supported by the role of the mRNA‐decapping subunit 1 (DCP1), which is a known mediator of pro‐inflammatory cytokine inhibition during sepsis.^14^ In addition, NUDT16 has recently been shown to mediate the selective degradation of Rift Valley fever virus mRNA, implicating the enzyme in immune response.^24^ The absence of articles among the sepsis literature relating to RNA decapping highlights a putative knowledge gap with regards to translational regulation of immune proteins, such as cytokines and chemokines, that are critical for sepsis development. Furthermore, the abundance of literature linking ADP ribosylation and inflammation provides additional avenues to explore how NUDT16 affects or is affected during the progression of an infection towards sepsis.

The role of NUDT16 in the context of sepsis or inflammation exhibits a knowledge gap and should be further explored experimentally. However, we showed that a joint interpretation of available literature and transcript profiling data conducted herein, along with basic experimental data, permitted inferences as to what role NUDT16 may play during inflammation and sepsis. We provided support for an indirect link between NUDT16 and inflammatory processes through ADP‐ribosylation and RNA decapping activities and infer the potential role for NUDT16 in the degradation of mRNAs and/or post‐translational regulation of inflammatory molecules during sepsis. These findings raise the question on what are the signalling pathways implicated in the modulation of NUDT16 expression in blood cells during sepsis.

## CONFLICT OF INTEREST

The authors confirm that there are no conflicts of interest.

## AUTHOR CONTRIBUTIONS

Susie **Shih Yin Huang:** Data curation (equal); Investigation (equal); Validation (equal); Visualization (equal); Writing – original draft (equal); Writing – review & editing (equal). **Darawan Rinchai:** Conceptualization (equal); Data curation (supporting); Investigation (supporting); Methodology (equal); Project administration (equal); Validation (supporting); Visualization (supporting); Writing – review & editing (supporting). **Mohammed Toufiq:** Data curation (equal); Investigation (equal); Software (equal); Validation (equal); Visualization (equal); Writing – review & editing (equal). **Basirudeen Syed Ahamed Kabeer:** Data curation (equal); Methodology (supporting); Validation (equal); Writing – review & editing (equal). **Jessica Roelands:** Data curation (equal); Validation (equal); Writing – review & editing (equal). **Wouter Hendrickx:** Resources (equal); Writing – review & editing (supporting). **Sabri Boughorbel:** Software (lead); Writing – review & editing (equal). **Davide Bedognetti:** Resources (supporting); Writing – review & editing (supporting). **Nicholas Van Panhuys:** Methodology (supporting); Resources (equal); Writing – review & editing (equal). **Damien Chaussabel:** Conceptualization (equal); Data curation (supporting); Investigation (supporting); Methodology (equal); Project administration (equal); Resources (equal); Validation (supporting); Visualization (supporting); Writing – original draft (equal); Writing – review & editing (equal). **Mathieu Garand:** Conceptualization (equal); Data curation (equal); Formal analysis (equal); Investigation (equal); Methodology (equal); Project administration (equal); Supervision (equal); Validation (equal); Visualization (equal); Writing – original draft (equal); Writing – review & editing (equal).

## ETHICAL APPROVAL

The study was approved by Sidra Medicine's Institutional Review Board. Written informed consents were obtained from the healthy donors.

## CONSENT FOR PUBLICATION

All the authors consent for publication.

## Supporting information

Fig S1‐S2

## Data Availability

The data that support the findings of this study are derived from the following resources available in the public domain: NCBI Gene Expression Omnibus (GEO) at [https://www.ncbi.nlm.nih.gov/geo/], reference numbers are mentioned in text.
