# Supplementary figures and images for: Transcriptomic profile investigations highlight a putative role for NUDT16 in sepsis
Source: J Cell Mol Med. 2022 Feb 17;26(5):1714–21. doi: 10.1111/jcmm.17240 (PMC8899167; doi:10.1111/jcmm.17240)

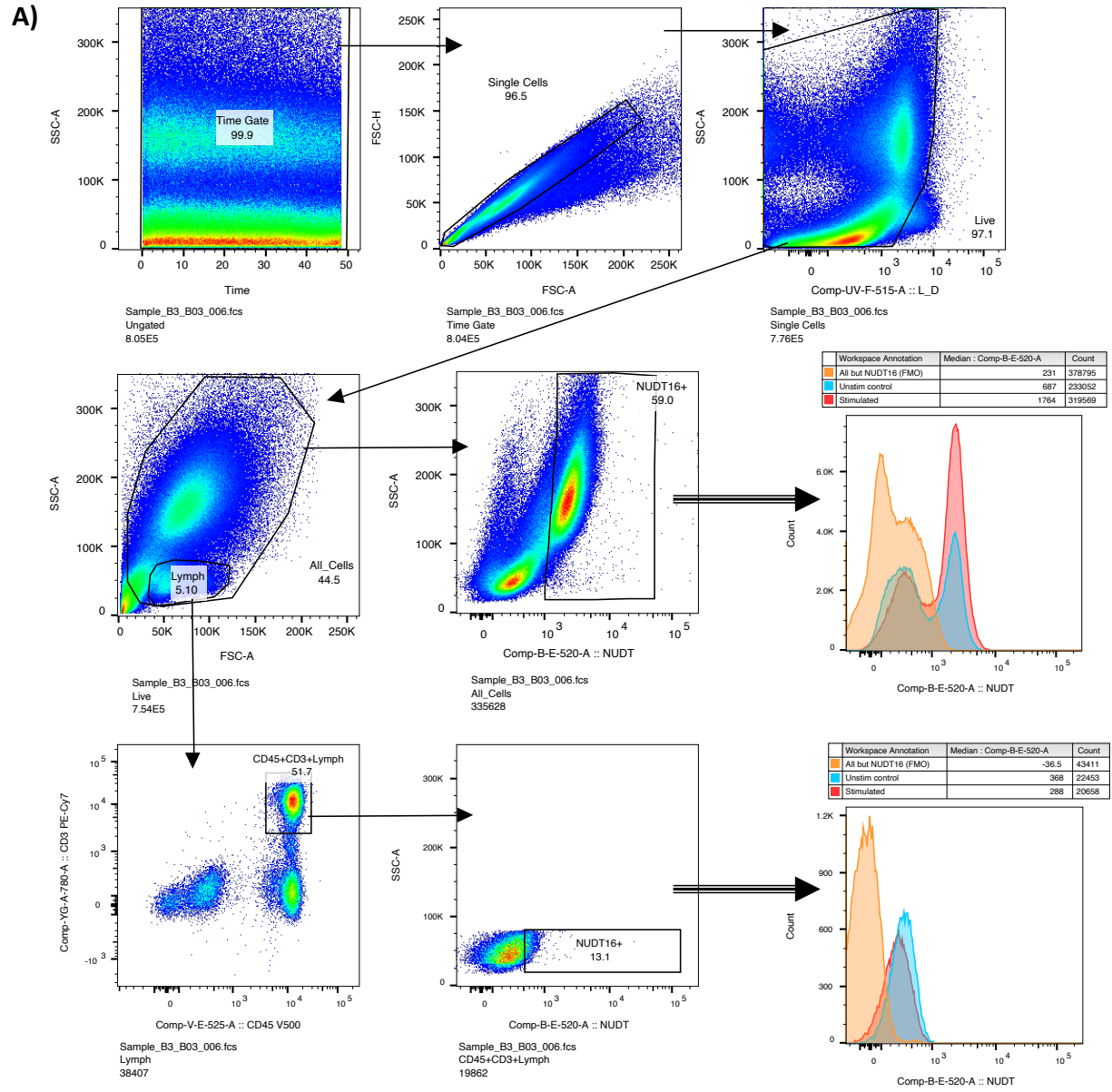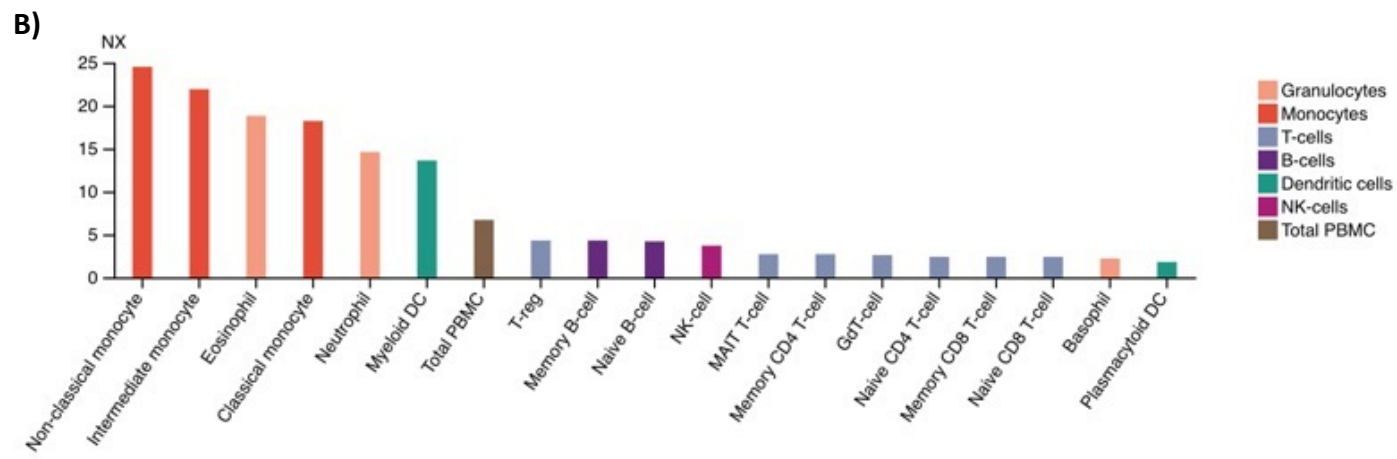

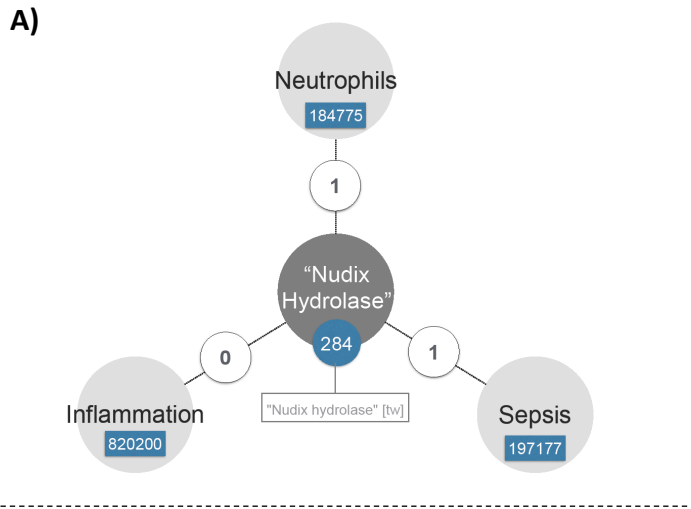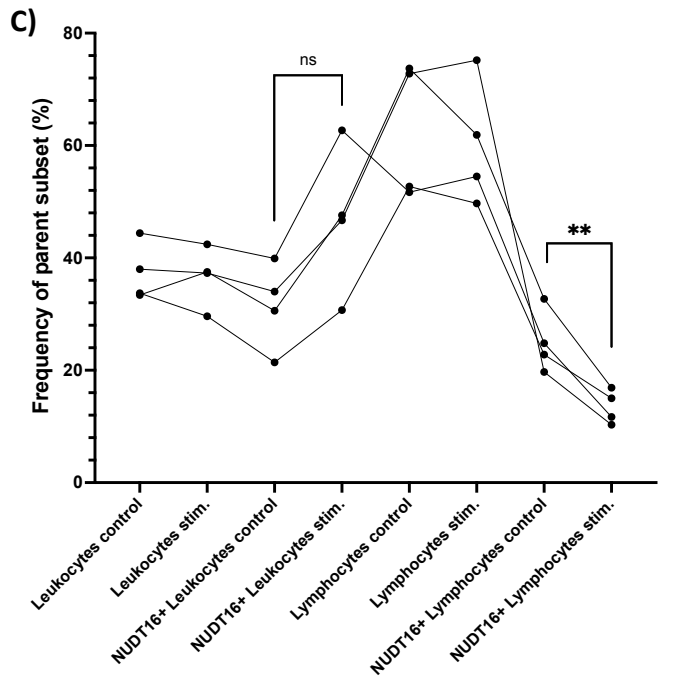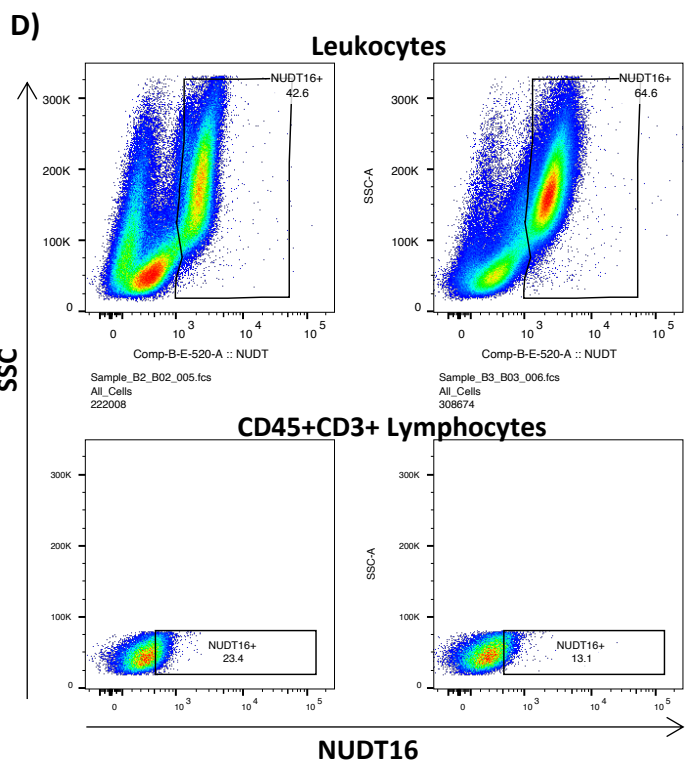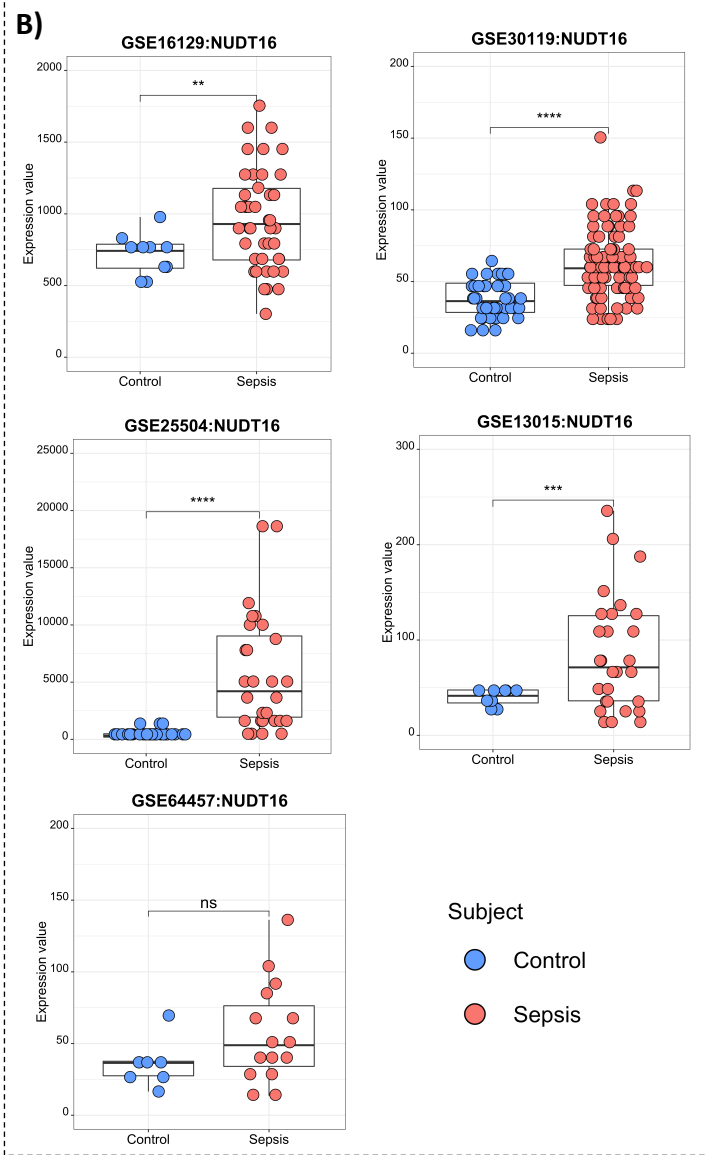

Supplement: Supplementary file 1 — Fig S1‐S2 [file JCMM-26-1714-s001.pdf]
